# Validation of Evidence-Based Fall Prevention Programs for Adults with Intellectual and/or Developmental Disorders: A Modified Otago Exercise Program

**DOI:** 10.3389/fpubh.2016.00261

**Published:** 2016-12-06

**Authors:** Mindy Renfro, Donna B. Bainbridge, Matthew Lee Smith

**Affiliations:** ^1^MonTECH/Rural Institute, University of Montana, Missoula, MT, USA; ^2^College of Public Health, Institute of Gerontology, The University of Georgia, Athens, GA, USA; ^3^Texas A&M School of Public Health, College Station, TX, USA

**Keywords:** falls, fall prevention, intellectual and/or developmental disorders, older adults, evidence-based program, Otago Exercise Program, functional performance

## Abstract

**Introduction:**

Evidence-based fall prevention (EBFP) programs significantly decrease fall risk, falls, and fall-related injuries in community-dwelling older adults. To date, EBFP programs are only validated for use among people with normal cognition and, therefore, are not evidence-based for adults with intellectual and/or developmental disorders (IDD) such as Alzheimer’s disease and related dementias, cerebral vascular accident, or traumatic brain injury.

**Background:**

Adults with IDD experience not only a higher rate of falls than their community-dwelling, cognitively intact peers but also higher rates and earlier onset of chronic diseases, also known to increase fall risk. Adults with IDD experience many barriers to health care and health promotion programs. As the lifespan for people with IDD continues to increase, issues of aging (including falls with associated injury) are on the rise and require effective and efficient prevention.

**Methods:**

A modified group-based version of the Otago Exercise Program (OEP) was developed and implemented at a worksite employing adults with IDD in Montana. Participants were tested pre- and post-intervention using the Center for Disease Control and Prevention’s (CDC) Stopping Elderly Accidents Deaths and Injuries (STEADI) tool kit. Participants participated in progressive once weekly, 1-h group exercise classes and home programs over a 7-week period. Discharge planning with consumers and caregivers included home exercise, walking, and an optional home assessment.

**Results:**

Despite the limited number of participants (*n* = 15) and short length of participation, improvements were observed in the 30-s Chair Stand Test, 4-Stage Balance Test, and 2-Minute Walk Test. Additionally, three individuals experienced an improvement in ambulation independence. Participants reported no falls during the study period.

**Discussion:**

Promising results of this preliminary project underline the need for further study of this modified OEP among adults with IDD. Future multicenter study should include more participants in diverse geographic regions with longer lengths of participation and follow-up.

## Introduction

Each year, approximately one in three older adults experiences a fall (1). Of those that fall, about 20% sustain fall injuries including (but not limited to) hip fracture and traumatic brain injury (TBI) (1). Evidence-based fall prevention (EBFP) programs have been shown to significantly decrease fall risk, falls (2), and fall-related injuries (3) among community-dwelling older adults with great return on investment (4). To date, these EBFP programs have been validated for use among people without cognitive impairment and, therefore, are not evidence-based for adults with intellectual and/or developmental disorders (IDDs) such as Alzheimer’s disease and related dementias (ADRD), cerebral vascular accident (CVA), or TBI.

The marginalized and vulnerable population of adults with IDD is rapidly increasing within the U.S. (5), as is the life expectancy for these individuals. Adults with IDD experience a higher rate of falls (6) relative to their peers who are community-dwelling and cognitively intact. Smulders et al. determined that the fall rate in this population was approximately threefold higher than their non-disabled peers (6). Although the fall circumstances and outcomes were similar, the falls rate among individuals with IDD was far higher. In addition, this population also shows higher rates and earlier onset of chronic diseases such as Type 2 diabetes (7), which may exacerbate fall risk (8). One particularly difficult risk factor for adults with IDD, especially Down syndrome, is the increased occurrence of ADRD (9). Secondary effects from chronic disease and mental health issues such as physical inactivity (10), social isolation and/or depression (11), and polypharmacy (12) are all known fall risk factors.

Adults with IDD experience many barriers (13) to healthcare and health promotion programs (14, 15). Provision of basic primary medical care, especially for women with IDD, may be difficult (16). Anderson et al. state that “people with intellectual and developmental disabilities (IDD) have experienced health disparities related to several factors including: a lack of access to high quality medical care, inadequate preparation of health care providers to meet their needs, the social determinants of health (e.g., poverty, race, and gender), and the failure to include people with IDD in public health efforts and other prevention activities” (17). In our Montana study, the IDD population experienced additional challenges due to rural access issues (18). Transitioning to adulthood can also present difficulties (19). Children with IDD often receive physical, occupational, and/or speech therapy through their schools up through age 18 or 21, depending upon state laws. Routine physical activity, therapy services, recreational experiences, and standing programs may not be available to these individuals (20) during the transition phase to adulthood and employment, which can negatively impact health, functional independence, and quality of life (21).

Increasing population size, much higher fall risk and fall rate, and barriers to preventive healthcare for adults with IDD combine to create a great and urgent need for expedient validation of EBFP programs tailored to meet the needs of this marginalized population. This community case study describes the promising results of a pilot intervention with potential to provide impetus for future intervention studies and funding priorities.

## Background and Rationale

Adults with IDD encompass a wide range of functional levels and challenges. According to the American Association on Intellectual and Developmental Disabilities (AAIDD), “*Intellectual disability* is a disability characterized by significant limitations in both intellectual functioning and in adaptive behavior, which covers many everyday social and practical skills. This disability originates before the age of 18” (22). IDD includes a large range of diagnostic categories including, but not limited to, autism spectrum disorders, some forms of cerebral palsy, Down syndrome, genetic disorders, toxic disorders, and traumatic disorders occurring during childhood. Medical definitions do not always coincide with state law definitions used to determine eligibility for services. For this community case study, great variations within the IDD population in terms of intellectual and physical functioning required selection of an EBFP that could be highly individualized and could still be offered in a group setting.

### Selection of Evidence-Based Fall Prevention Program

The research team considered many EBFP programs for validation in this population. The best program would have the highest tier evidence with well-documented outcomes to decrease fall-related risk, fall rates, and/or fall-related injuries (23). The best program would also have the capability to be widely disseminated in many geographic areas of the U.S. In addition, easily accessible training modules were necessary to close the gap between knowledge and dissemination (24). The selected EBFP would be easily adaptable to wide variations in physical and cognitive functioning and include exercise for lower extremity strength and standing balance, both known to improve fall risk for the population (25). We placed a heavier emphasis on physical activity than on education because educational components might present difficulties to translation and teaching for this population. The selected EBFP needed to be easily translated into an effective home exercise program to achieve 50 h/6 months or 2 h/week at a minimum, of progressively challenging balance exercise to improve balance (26). In addition, the selected EBFP would offer flexibility to individualize the exercises based upon the participants’ current balance and strength.

Following review of over 25 EBFP programs, the principal investigators’ (PIs) final decision was to utilize the Otago Exercise Program (OEP). OEP is a menu-driven individualized program delivered one-to-one by a physical therapist (PT) to one patient in the home setting with a method for independent progression of the exercises to increase challenge and outcomes (27). The advantages of OEP include, but are not limited, to the following: it is highly individualized; it has a focus on physical activity; and it includes limited educational material and/or didactic learning. PTs can be trained online as OEP leaders at a nominal cost, and training is designed for ongoing independent program use and progression. The disadvantage in using OEP for this project is that OEP is usually offered one-to-one using billable PT services, which limits access to service and dissemination of OEP to this marginalized population. OEP leader training is focused on PTs and PTAs only in the U.S. (28); however, nurses in New Zealand and Australia offered OEP successfully.

The research team felt that OEP was the best program to provide with the least amount of modification to meet the diverse needs of the IDD population. We modified the program for this population, offering group programs with individualized programing. OEP allows for individualized intervention based upon variations in physical functioning and independence. Given the varied functional levels of the adults with IDD, OEP was a natural selection. These group programs improved social interaction while serving more people with less staff and limited travel time.

### Selection of Community Partner

The PIs looked for a community partner who provided services to a group of adults with IDD within an accessible, centrally located building that provided ease of access for the potential participants, participating students, and PIs. The facility would need to provide an exercise room that was of adequate size, safety, and was available at various times convenient for use (including an administration willing to participate in this weekly project over a 10-week period). Many providers and fitness centers were considered and approached. Opportunities Resources, Inc. (ORI) met all of the project’s needs and was a willing partner (29). ORI employs over 350 trained professionals who provide employment, living skills, and support, plus a broad array of educational, recreational, and companion services to nearly 700 adults with disabilities, as well as case management to over 1,500 persons with IDD, mental illness, physical disabilities, and/or TBI. ORI was able to provide their fitness room to provide classes and a meeting room for pre- and posttesting.

## Essential Elements of the Intervention

Study PIs designed the project to meet the unique needs of the population, received Institutional Review Board (IRB) approval, and shared the program with potential community partners and on-campus academic health programs (for participant recruitment purposes).

### Participants

This research project was approved by the IRB of the University of Montana (IRB # 235-15) prior to any participant recruitment. Participants to be included were individuals with IDD aged 18 years or older who lived at home or in supported community environments. The investigators contacted ORI, who was interested in engaging in this project and arranged a meeting with their supported living staff. The investigators explained the OEP, the project, and the inclusion/exclusion criteria. Staff members were provided the opportunity to become OEP certified *via* the online training program. https://www.med.unc.edu/aging/cgec/exercise-program.

Participants who met inclusion criteria and were interested in the program were requested to sign a Participant Information and Consent that had been approved by the IRB. Trained research staff or supervised students explained the project and the consent form to each participant. Participants who consented were also asked to sign an authorization to use and disclose medical information for research purposes so we could access their medical histories. If the participant was not their own guardian, the legally designated representative was engaged to sign both forms. Once these forms were signed, participants were given an identifying number for confidentiality prior to starting the program. The project had 18 initial participants. One participant completed pretesting, then dropped from the program, one completed pre-post testing, but not the program, and one participant did not complete post-testing. Thus, we had 15 active participants in the program. Participants (male = 5; female = 10) were between the ages of 27 and 70 years. The majority of the participants had a diagnosis of IDD. One participant had mental illness, and one participant had a TBI. Six participants (one with TBI, one with mental illness, and four with IDD) also had physical disabilities including hemiparesis, arthritis, and cerebral palsy.

#### Program Instructors

One or both of the PIs were present at all testing and class sessions. Students from the University of Montana’s School of Physical Therapy and Rehabilitation and the Montana State University-Bozeman’s School of Nursing programs were offered the opportunity to assist with testing and class instruction as part of their research and/or public health coursework. All participating students were required to complete the CITI Human Subjects Protection Training Course and the online OEP training from the University of North Carolina’s School of Medicine prior to the start of the study. Seven nursing and five physical therapy students assisted with the project. Occasional visits from nursing and PT faculty increased supervision and monitor fidelity.

#### Testing Protocols

Two afternoon sessions for pre- and post-intervention testing were scheduled. Each participant completed the following: Stopping Elderly Accidents, Deaths, and Injuries (STEADI) stay independent questionnaire (30) and a FallPAIDD personal function survey (31). Support staff provided assistance to participants as needed. The functional screens administered were those outlined in the STEADI (32) and included orthostatic hypotension (33–35), Timed Up-and-Go (TUG) Test (36–38), 30-Second Chair Stand Test (39–42), and the 4-Stage Balance Test (13, 43–45). The PIs added the 2-Minute Walk Test as a method to determine aerobic ability of participants (46–48). Given that there was not a detailed PT evaluation completed for these participants, test results were helpful in determining a starting level for OEP classes.

#### Functional Assessments

The Timed-Up-and-Go Test is designed to test mobility skills, balance, and fall risk in older persons. The test requires that a person stand from a chair, walk 10 ft, turn, walk back to the chair and sit. Higher fall risk is defined as >12 s to complete the TUG. Decreased time for the test at post-testing indicated improvement.

The 30-Second Chair Stand Test assesses lower extremity strength and endurance. The person crosses his arms on his/her chest, then stands and sits repetitively for 30 s. Use of hands to stand is a zero score. A greater number of rises in 30 s at post-testing indicated improvement.

The 4-Stage Balance Test is an assessment of static balance in four different and increasingly challenging positions – feet together, instep of foot advanced to toe of other foot, foot in front of other foot (tandem), and single leg stance. Success is maintenance of each position for 10 s; less than 10 s indicates stage failure. Passing is completion of the third stage for 10 or more seconds. Improvement was demonstrated by completion of more test stages (0–4) at post-testing.

The 2-Minute Walk Test is a submaximal assessment of basic aerobic ability. The participant is requested to walk as fast as they can for a period of 2 min, and the distance covered in that period is recorded. Improvement was demonstrated by coverage of more distance in the 2-min period at post-testing.

#### OEP Program and Adaptation

The OEP as adapted for use in the U.S. is a home-based one-on-one program conducted by an OEP-trained PTs as part of a full treatment plan for improvement of strength, balance, and fitness to reduce falls in frail older adults (28). As designed, it is a 12-month program consisting of seven in-home visits, seven telephone contacts, and monthly monitoring of exercise compliance and falls. Close to the completion of the OEP, the PT refers the patient to an appropriate community-based program for continuation of fall prevention education and exercise.

For this project, we focused on maintaining program fidelity as much as possible, making alterations only as necessary for the study population. Characteristics of the OEP that were unchanged from the original program included certification of all leaders (including students) in OEP, use of the OEP outline/menu of exercises, pre-participation query of medical issues, weekly treatment time of 50–60 min including warm-up, exercise and walking components, and use of home practice exercises with weekly logs (including fall reports). Attendance and participation was excellent (>90%) over the 7 weeks of class sessions. Classes were held in the fitness room, providing a safe and enclosed exercise environment. Each of the two 1-h classes averaged 8 participants, 2–3 caregivers/staff, 5–7 nursing students, and 1 or 2 PI/PTs. At the conclusion of the program, a home exercise program was prescribed for each participant and a home assessment was offered.

Characteristics of the OEP that were modified for this population included solicitation of clients without PT referral, addition of pre- and posttests from the CDC’s STEADI tool kit, use of the OEP in a group setting rather than one-to-one, and a much shorter length of intervention (7 weeks in this project as opposed to 6–12 months in standard OEP). As with the general population, it is challenging to promote adherence to home programs among adults with IDD (49). Research documents the efficacy of group or “buddy” programs in this population for motivation and adherence. The group setting also promotes social inclusion (50–56).

#### Statistical Analyses

All data analyses were performed using SPSS (version 24). Frequencies were calculated to identify participant characteristics. Descriptive statistics were calculated for functional assessments at baseline and follow-up. Paired sample *t*-tests were used to identify the magnitude of functional assessment changes over time as a result of the intervention. Wilcoxon sign-rank tests were used to identify the proportion of participants who improved their functional assessment scores over time as a result of the intervention. A series of repeated measures ANOVA were performed to assess significant changes in the dependent variable, controlling for age group (18–49 vs. 50+ years) and then sex (female vs. male). The authors did not perform a power analysis prior to conduct this exploratory pilot study; therefore, results of a power analysis are not reported in the manuscript. Considering the small sample size utilized in this pilot study, the authors used α = 0.2 as a less conservative criterion in all analyses to identify relationships determined to be approaching statistical significance.

## Results

### Sample Characteristics

Of the 15 participants, 46.7% were age 50 years and older. Approximately, 67% of participants were females and 93.3% reported one or more chronic condition diagnosis (see Table 1).

**Table 1:** **Sample characteristics (*n* = 15)**.

**Age group**	
18–29	6.7%
30–39	13.3%
40–49	33.3%
50–59	13.3%
60–69	26.7%
70+	6.7%
**Sex**	
Male	33.3%
Female	66.7%
**Chronic condition**	
No	6.7%
Yes	93.3%

### Magnitude of Change

On average from baseline to follow-up, participants increased from 8.60 to 10.27 rises in the 30-Second Chair Stand Test. The trend of this change was approaching significance (*t* = −1.60, *P* = 0.132). On average from baseline to follow-up, participants increased from 114.43 to 194.62 steps in the 2-Minute Walk Test. The trend of this change was significant (*t* = −3.80, *P* = 0.002). On average from baseline to follow-up, participants advanced their ability to perform greater on the 4-Stage Balance Test (from 1.87 to 2.20 out of 4 stages). The trend of this change was approaching significance (*t* = −2.09, *P* = 0.055) (see Table 2).

**Table 2:** **Paired sample *t*-tests (*n* = 15)**.

	Baseline mean (SD)	Follow-up mean (SD)	*t*	*P*
30-Second Chair Stand Test (rises)	8.60 (±3.78)	10.27 (±4.28)	−1.60	0.132
Timed Up-and-Go (TUG) Test (s)	17.62 (±5.28)	16.36 (±5.86)	1.28	0.220
2-Minute Walk Test (m)	114.43 (±45.41)	194.62 (±92.78)	−3.80	0.002
4-Stage Balance Test (range 0–4)	1.87 (±0.83)	2.20 (±0.77)	−2.09	0.055

### Proportion of Participants Showing Improvement

When examining the proportion of participants who improved their functional assessment scores, 53.3% of participants improved on the 30-Second Chair Stand Test. The change in this proportion of participants was approaching significance (*z* = −1.42, *P* = 0.156). Approximately 67% of participants improved on the 2-Minute Walk Test. The change in this proportion of participants was significant (*z* = −2.54, *P* = 0.011). Approximately 27% of participants improved on the 4-Stage Balance Test (the remaining 73.3% remained the same from baseline to follow-up). The change in this proportion of participants was approaching significance (*z* = −1.89, *P* = 0.059) (see Table 3).

**Table 3:** **Wilcoxon sign-rank tests (*n* = 15)**.

	Negative	Positive	Ties	*Z*	*P*
30-Second Chair Stand Test (rises)	4	**8**	3	−1.42	0.156
Timed Up-and-Go (TUG) Test (s)	**10**	5	0	−1.28	0.201
2-Minute Walk Test (m)	4	**10**	1	−2.54	0.011
4-Stage Balance Test (range 0–4)	0	**4**	11	−1.89	0.059

*Bold values indicate the number of participants who improved from baseline to post-intervention*.

### Rate of Change by Age

**Figure 1:**
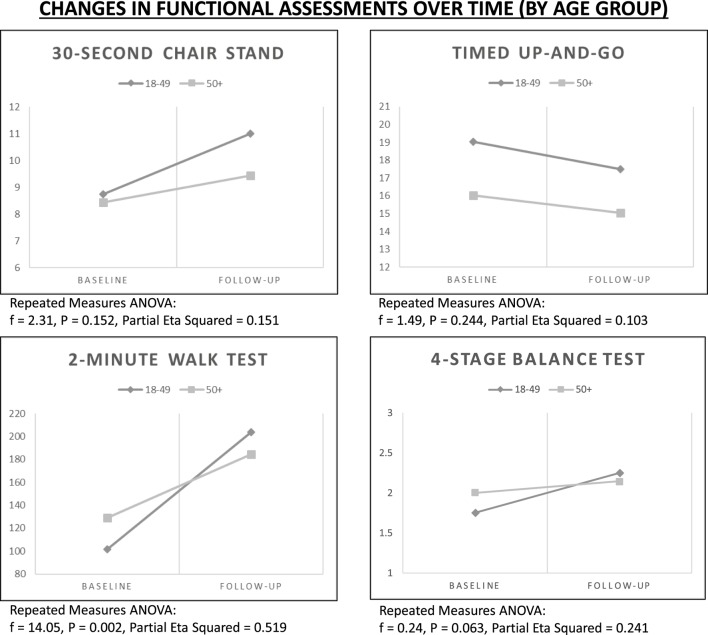
**Changes in functional assessments over time by age group**.

Using repeated measures ANOVA, improvements in functional assessments were examined over time controlling for age group (see Figure 1). Despite general gains for the 30-Second Chair Stand Test, rates of improvement were more dramatic for participants in the younger age group (*f* = 2.31, *P* = 0.152). Despite general gains for the 2-Minute Walk Test, rates of improvement were more dramatic for participants in the younger age group (*f* = 14.05, *P* = 0.002). Despite general gains for the 4-Stage Balance Test, rates of improvement were more dramatic for participants in the younger age group (*f* = 0.24, *P* = 0.063).

### Rate of Change by Sex

**Figure 2:**
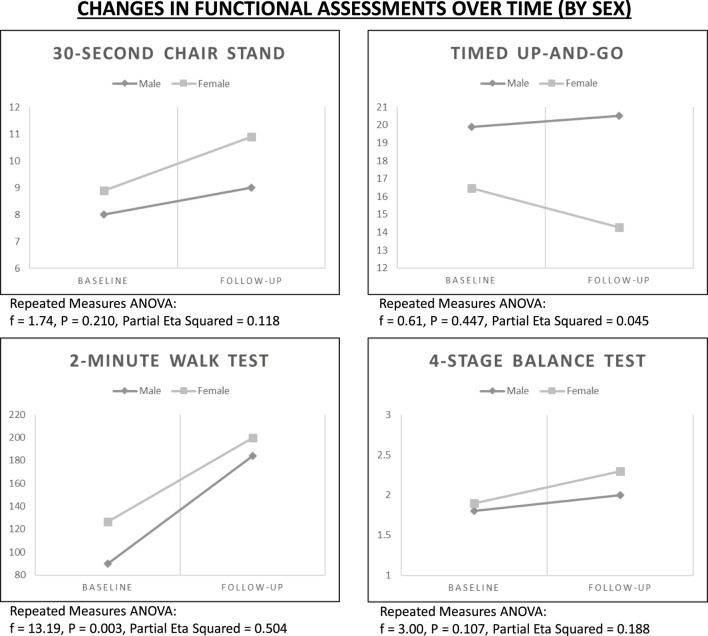
**Changes in functional assessments over time by age sex**.

Using repeated measures ANOVA, improvements in functional assessments were examined over time controlling for sex (see Figure 2). Despite general gains for the 2-Minute Walk Test, rates of improvement were more dramatic for male participants (*f* = 13.19, *P* = 0.003). Despite general gains for the 4-Stage Balance Test, rates of improvement were more dramatic for female participants (*f* = 3.00, *P* = 0.107).

## Discussion

This pilot project demonstrated that the utilization of the OEP in adults with IDD produced test results that were similar to results to older adults with no cognitive impairments (57–60). The positive trends toward improved strength (30-Second Chair Stand Test), balance (4-Stage Balance Test), and aerobic ability (2-Minute Walk Test) over the course of the program occurred as noted in participants with no impairments in cognition. These results indicate that the OEP has potential as a successful programmatic way to decrease the risk of falls among adults with IDD.

In this study, changes were generally greater for the younger group of participants <50 years, suggesting that reduction of fall risk can occur at an earlier age among adults with IDD, which is important considering these individuals age at and experience falls risk at different rates of those without IDD (6, 7, 61). These results suggest that early initiation of programing to reduce the risk of falls in both groups is a beneficial idea.

More importantly, the modifications of the OEP for adults with IDD did not negatively impact the outcomes of the program. Changes were still observed, despite modifying the OEP to a group program that was only 7 weeks in length and used exercise bands instead of weights for resistance. Participants demonstrated improvements in characteristics that would lower fall risk (lower extremity strength, balance, and aerobic conditioning). Over 50% of participants improved their strength, while 67% improved aerobic ability. A smaller percentage (27%) demonstrated improvements in balance, but these results may represent the physical disabilities of the IDD participants, not necessarily the OEP translation.

These results suggest that maintaining veracity in the key elements of the program (e.g., utilization of the specific exercises and routines, home exercise programs with logging of performance, and individualization of programing) are the vital to program integrity. Other aspects of the original OEP may not be necessary to evoke meaningful changes among participants (e.g., one-on-one intervention, type of resistance, or specific professional instructor). The larger implication is that modification and translation to new populations is possible with fidelity to these key program elements, which opens the program utilization to many more population groups (e.g., those with dementia, TBI).

### Implications for Practice

This project presented several strong implications for practice. First, development of partners is vital to program success. A dedicated partner who can facilitate recruitment of participants and perhaps provide exercise space is the key. Their clients will be more interested and amenable to participation if they feel the organization is supportive, and if they feel comfortable in familiar space that is convenient to their daily lives.

Second, although supervision of a PT is essential to OEP activity progression during the intervention and training of class leaders, other professionals may be well suited to lead the group classes (e.g., PT assistant, medical assistant, and nurse) (62). These allied health professionals may benefit from the experience of hosting OEP with IDD participants, and being exposed to use of progressive balance exercises. Such experience can teach professionals the importance of balance for various populations and demonstrate how best to improve balance and educate population groups who have cognitive impairments with concomitant physical disabilities.

The experience of this project highlighted the possibility of the effective use of peer leaders in a specific population (63). As the IDD participants became more engaged with the leaders and their fellow participants, several stepped forward to either count or lead exercises during each class, which was met with the enthusiasm of their peers.

Third, because of the unique needs of adults with IDD, this pilot study reinforced the notion of starting to work on balance and falls risk early. We noted greater improvements among our younger participants with IDD (<50 years); therefore, implementing the intervention at an earlier age might reduce fall risk, delay the onset of falls, and reduce the overall number of falls.

Finally, the key to maintaining low fall risk is the use of ongoing balance exercise that is progressively more challenging. Hence, the issue of program sustainability is vital. Developing infrastructure including staff support can maintain the programing and provide internal sustainability for clients. Program internalization can further improve client safety by lowering fall risk and reducing falls in their daily and living environments. Online training in OEP can be supplemented by additional tailored training, *via* class, telehealth, or webinar, to address the specific issues of clients with intellectual disabilities. Finally, providing ongoing mentorship and consultation by PT professionals also creates a resource for staff.

### Implications for Research

Major strengths of this project were its positive outcomes and potential for replicability. However, this pilot project had several weaknesses, which present implications for future research. First, the project had a small number of participants, so it may have been underpowered to detect all significant changes among participants. We recommend that future studies replicate the program with larger numbers of participants and with more diversity (e.g., age, IDD diagnoses, and comorbidities) to validate findings of this community case study and advance the literature/knowledge in this area.

Second, the course of this program was quite short (7 weeks). Replication of the program over a longer period would assist with defining a minimum program length capable of yielding significant improvements among participants. Further, collecting follow-up data after a longer period would enable researchers to determine if initial intervention effects are maintained over time and whether more tangible outcomes can be seen (e.g., number of falls, fall injury rates, and medical costs).

Third, the addition of other process and outcome measures has the potential to increase what is known about broad spectrum fall prevention programs for adults with IDD. Currently, there is little research about the built environment of IDD participants’ home and work, which has potential to increase or suppress falls risk among this population. Research indicates that safety and “fit” of the built environment are factors that can reduce falls (64). Beyond environmental fit, exploration of the support needed for maintenance of maximum independence of this population in their environment is indicated.

## Conclusion

This community case study demonstrated that the OEP can be successfully modified and conducted with adults with IDD. More research is needed to determine the effect of this translation on fall rates and costs as well as long-term sustainability of the intervention’s effectiveness in adults with IDD. The positive results of this pilot study is a necessary first step toward fall risk reduction among a marginalized population of adults with IDD, having significantly greater and earlier onset falls burden. A future multicenter trial of longer duration is needed to advance this research.

## Author Contributions

MR, DB, and MS wrote the manuscript. MR and DB designed the study and collected the data. MS performed statistical analyses.

## Conflict of Interest Statement

The authors declare that the research was conducted in the absence of any commercial or financial relationships that could be construed as a potential conflict of interest.
